# The Influence of Pore Size on the Indentation Behavior of Metallic Nanoporous Materials: A Molecular Dynamics Study

**DOI:** 10.3390/ma9050355

**Published:** 2016-05-11

**Authors:** Daniel Esqué-de los Ojos, Eva Pellicer, Jordi Sort

**Affiliations:** 1 Departament de Física, Universitat Autònoma de Barcelona, Bellaterra E-08193, Spain; danielesque@gmail.com (D.E.-d.l.O.); eva.pellicer@uab.cat (E.P.); 2 Manchester Materials Science Centre, The University of Manchester, Grosvenor Street, Manchester M1 7HS, UK; 3 Institució Catalana de Recerca i Estudis Avancats (ICREA), Barcelona E-08010, Spain

**Keywords:** elastic properties, molecular dynamics, spherical indentation, porous materials

## Abstract

In general, the influence of pore size is not considered when determining the Young’s modulus of nanoporous materials. Here, we demonstrate that the pore size needs to be taken into account to properly assess the mechanical properties of these materials. Molecular dynamics simulations of spherical indentation experiments on single crystalline nanoporous Cu have been undertaken in systems with: (i) a constant degree of porosity and variable pore diameter; and (ii) a constant pore diameter and variable porosity degree. The classical Gibson and Ashby expression relating Young’s modulus with the relative density of the nanoporous metal is modified to include the influence of the pore size. The simulations reveal that, for a fixed porosity degree, the mechanical behavior of materials with smaller pores differs more significantly from the behavior of the bulk, fully dense counterpart. This effect is ascribed to the increase of the overall surface area as the pore size is reduced, together with the reduced coordination number of the atoms located at the pores edges.

## 1. Introduction

Owing to their high surface area, interest in the use of porous materials for different technological applications (chemistry [1,2,3,4], optics, electronic and magnetic devices [5,6], biomedicine [7], *etc.*) has tremendously increased during the last few years. Porous materials can be classified into macro-, meso- and nanoporous, depending on their pore size. When it comes to metals, one can distinguish between cellular metals (having a large fraction of porosity, typically beyond 70%) and metal foams (having pores deliberately integrated into their structure through a foaming process [8]). Metal foams can exhibit closed or open cells in either ordered or random spatial arrangement [9,10]. The term “nanofoam” has been recently adopted to refer to metals having pores of a few nanometers in diameter. In all these cases, determination of the mechanical properties is of uppermost importance to assess the structural integrity of these materials. With this aim, several studies have focused on the determination of Young’s modulus and its correlation to the material’s porosity degree. For example, Murillo *et al.* [11] studied the evolution of the Young’s modulus of silica aerogels with their density by recourse to molecular dynamics (MD) simulations of tensile experiments. Another example is the work by Cohen-Tanugi and Grossman [12], who studied the evolution of Young’s modulus with porosity in a nanoporous graphene sheet loaded under biaxial stress. However, to the authors’ knowledge, while most of the models take the porosity degree properly into account, the influence of pore diameter is generally overlooked. In general, the scientific community has used the expression given by Gibson and Ashby [9] to relate Young’s modulus and yield stress with the porosity degree of the material under study:

(1) $$E^{*}=C_{2}E_{s}{\left(\frac{\rho^{*}}{\rho_{s}}\right)}^{n}$$

where E* and *E*_s_ are the Young’s moduli of the porous and bulk materials, respectively; *C*_2_ is usually taken to equal 1; *n* = 2 for open-cell foams, and *n* = 3 for close-cell foams [9]. A similar expression applies for the yield stress:

(2) $$\sigma^{*}=C_{2}^{\prime }\sigma_{s}{\left(\frac{\rho^{*}}{\rho_{s}}\right)}^{m}$$

where σ* and *σ*_s_ are the yield strengths of the porous and bulk materials, respectively; the coefficient $C_{2}^{\prime }$ is equal to 0.3; and *m* = 3/2 for open-cell foams, whereas *m* = 1 for close-cell foams. These equations were derived considering an idealized cell geometry together with data on mechanical tests from macropore size foams. Hence, these scaling equations have been shown to satisfactorily describe the mechanical behavior of macrocellular foams [13]. However, in the case of nanoporous foams, the agreement between the measured mechanical properties and the ones predicted from the Gibson and Ashy’s equations is not as good [14,15]. To improve the agreement, a drastic increase of the yield stress at the nanoscale has to be considered [14], or the equations of Gibson and Ashby need to be modified to include the influence of the ligament size [15]. Previous computational approaches have consisted in studying the collapse of a single nanovoid or a collection of nanovoids in face-centered cubic (fcc) and body-centered cubic (bcc) metals [15,16,17,18,19], focusing on the dislocations activity accompanying the pores collapse.

A previous work by Yuan and Wu [20] studied the effects of the relative density and the pore diameter on the adiabatic uniaxial compression behavior and the related atomic-level deformation mechanisms in nanoporous copper using MD simulations. Herein, we demonstrate that the pore size itself plays a key role in the resulting mechanical behavior of metallic nanofoams during nanoindentation, and we introduce a formalism to take it into account in the analytical modeling of the mechanical properties of these materials.

## 2. Simulation Methods

Molecular dynamics (MD) simulations were performed by means of the Large-scale Atomic/Molecular Massively Parallel Simulator LAMMPS code [21] using the embedded atom model (EAM) developed by Mishin *et al.* [22] for modeling of spherical nanoindentation experiments on Cu crystals with the (001) crystallographic oriented plane. The size of the simulation box was 67 nm × 42 nm × 67 nm, where the thickness is equal to 42 nm. Periodic boundary conditions were imposed on the lateral sides of the simulation box to prevent its rotation during the indentation process, while a layer of atoms at the bottom was frozen to prevent vertical displacement of the simulation box. The spherical indenter was modeled through a quadratic repulsive potential as in [23,24,25], with a diameter of 24 nm. Indentation velocity was kept constant and equal to 4 m/s for all simulations. Previous to indentation, simulation box was relaxed at a temperature of 77 K to minimize thermal effects. In order to study the influence of the pore diameter on the elastic behavior of the indented material, we compared the Young’s modulus resulting from the indentation of bulk Cu (containing more than 16 millions of atoms according to the aforementioned dimensions of the simulation box) with systems having: (i) a constant degree of porosity equal to 15% and pore diameters equal to 2, 4, and 8 nm with ligament sizes equal to 1, 2, and 4 nm, respectively; and (ii) a constant pore diameter *D*_p_ = 8 nm and variable degree of porosity (5%, 10%, 15%, 20%, and 45% with ligament sizes equal to 12, 6.5, 4, 2.5, and 0.5 nm, respectively). The geometry of the pores was spherical, and the pores were placed inside the simulation box uniformly following a cubic reticule. Even though one could simply perform traction or compression experiments in order to study the effect of porosity on Young’s modulus, here, we decided to use MD spherical indentation simulations to study the gradual effect that porosity can cause on the nanomechanical properties of this nanoporous material. The simulation geometry resembles experiments where an atomic force microscopy (AFM) tip could be used to indent a porous single crystal.

## 3. Results and Discussion

**Figure 1:**
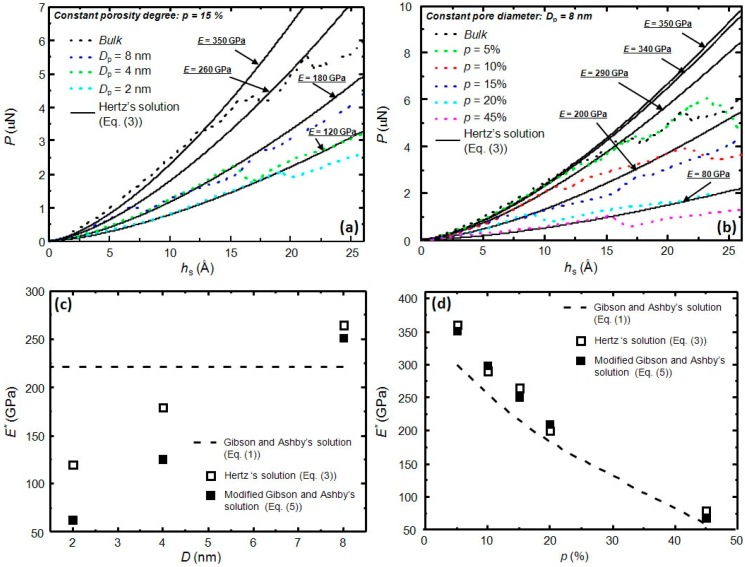
(**a**) Applied load (*P*)—penetration depth (*h*_s_) curves for molecular dynamics (MD) simulations (dotted lines) of spherical indentation on (001) copper single crystals. Solid lines correspond to Hertz’s solution (Equation (3)) for spherical indentation, used to determine the elastic moduli (indicated in the plot) during the first stages of indentation for the different simulations. Except for the bulk case, all simulation boxes in (**a**) correspond to a constant porosity *p* = 15% and a variable pore diameter (*D*_p_), from 2 to 8 nm; (**b**) Same as in (**a**), but here, except for the bulk case, all simulation boxes correspond to a constant pore diameter *D*_p_ = 8 nm and a variable porosity degree (*p*), ranging from 5% to 45%; (**c**) Dependence of Young’s modulus (E*) on the pore diameter (*D*_p_) for a constant porosity *P* = 15%. The plot compares the elastic moduli extracted from MD simulations using Equation (3) (Figure 1a) with the predicted values obtained from Equation (1) and with the proposed modification of the Gibson and Ashby relation (Equation (5)); (**d**) Dependence of Young’s modulus (E*) on the porosity degree (*p*) for a constant pore diameter (*D*_p_ = 8 nm). Again, the plot compares the elastic moduli extracted from MD simulations (Figure 1b) with the predicted values obtained from Equation (1) and with the proposed modification of the Gibson and Ashby equation (Equation (5)).

Figure 1a,b shows the applied load (*P*)—penetration depth (*h*_s_) curves obtained from MD simulations for constant porosity and constant pore diameter, respectively. To determine the elastic modulus of each curve, we used the Hertz solution that predicts the elastic contact during spherical indentation [26]. Each simulation curve is accompanied with its corresponding Hertz solution that follows this expression:

(3) P=Ch32

where *C* is a constant that depends on the diameter of the spherical tip (*D*) and the elastic modulus *E* of the indented material, according to:

(4) C=223ED12

By fitting the MD simulations results with Equation (3) at the first stages of indentation (*i.e.*, before dislocation nucleation), one can determine the elastic modulus of each curve. From Figure 1a, it is worth noting that, for a given porosity of 15%, the curve closer to the bulk behavior is the one corresponding to the biggest pore diameter, *D*_p_ = 8 nm. Reducing the pore size, while keeping a constant porosity degree, has the net effect of reducing the elastic modulus (see Figure 1c). Remarkably, this effect is not captured by Equation (1). Another unexpected result is the high elastic modulus found for bulk Cu that, with a value of 350 GPa, highly differs from the 120 GPa commonly reported for bulk polycrystalline Cu. Fang *et al.* [27] already reported values similar to the present one in a simulated nanoindentation experiment (283.4–444.9 GPa), proposing that such a large discrepancy could be related to scale differences between experiments and simulations (nano-scale *vs.* micro-scale), stemming from the different influence of the defect concentration on the deformation mechanism of the material at the two different scales. A pronounced increase of the Young’s modulus in different types of nanomaterials has also been observed experimentally using different techniques, such as bending [28], *in-situ* compression in transmission electron microscopy [29], AFM [30], *etc*.

Figure 1b shows the expected behavior of the load—displacement curves as a function of porosity degree. Namely, for a given pore size *D*_p_ = 8 nm, increasing the porosity leads to a reduction of the load required to attain a given penetration depth, *i.e.*, a reduction in the elastic modulus.

As aforementioned, the expression from Gibson and Ashby [9] is commonly used to extract the elastic modulus of a porous materials (E*) provided that the porosity degree and the elastic modulus of its bulk counterpart are known. From Equation (1), one can see that pore size is not considered for the determination of E*. Here, we introduce a modification to Equation (1) in order to take the pore size into account. The pore size is considered as an scaled parameter, in relation to the diameter of the indenter tip used during simulations (*D*_i_ = 48 nm):

(5) $$E^{*}=C_{2}E_{s}\left[a_{1}\left(\frac{D_{p}}{D_{i}}\right)\right]{\left(\frac{\rho^{*}}{\rho_{s}}\right)}^{n}$$

where *a*_1_, *C*_2_, and *n* are fitting parameters. We used the elastic moduli values derived from the adjustment of the simulated *P*—*h*_s_ curves in Figure 1 with Equation (3) to determine the set of values for the above parameters in Equation (5), resulting in *C*_2_ = 1, *n* = 3, and *a*_1_ = 7. It is worth noting here that the values used for *C*_2_ and *n* in Equation (5) are the same as the ones used in Gibson and Ashby [9] for close-cell foams, as in the pores arrangement of our present study. Figure 1c,d shows the comparison between the fitted and the calculated values for the elastic moduli. Even though the agreement is not perfect for a constant degree of porosity (Figure 1c for *p* = 15%) the modified expression of Gibson and Ashby (Equation (5)), shows to capture quite accurately the evolution of the Young’s modulus for a fixed pore size *D*_p_ = 8 nm and variable porosity (see Figure 1d). The agreement is much worse if one uses Equation (1) (instead of Equation (5)) to assess the Young’s modulus of the indented porous material (see both Figure 1c,d).

**Figure 2:**
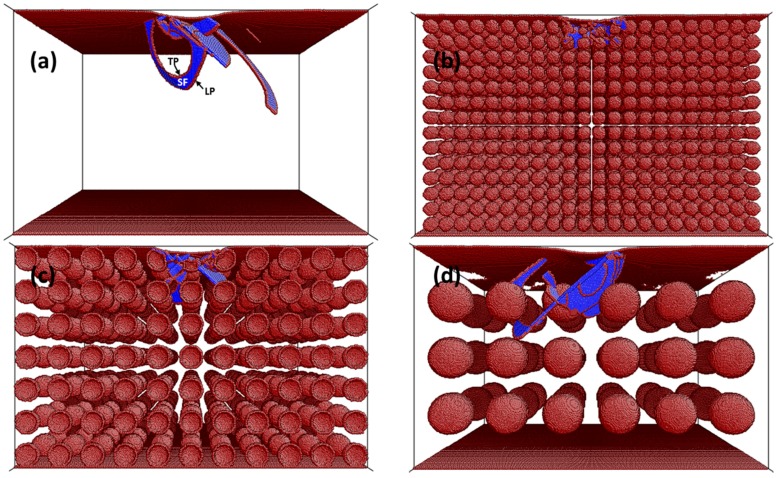
Snapshots showing the dislocation structure at a constant penetration depth (*h*_s_ = 15 Å) of spherical MD indentation simulations on (001) Cu single crystals for: (**a**) bulk; (**b**) *D*_p_ = 2 nm; (**c**) *D*_p_ = 4 nm; (**d**) *D*_p_ = 8 nm. The porosity level in panels (**b**–**d**) is fixed to *p* = 15%. Panel (**a**) shows the stacking faults (SFs) delimited by leading partial (LP) and trailing partial (TP) dislocations. Dark red color designates atoms with a reduced coordination number.

In order to understand why, for a fixed porosity, larger pores result in a behavior closer to that of the bulk material (as shown in Figure 1a), the dislocation structures produced during indentation were visualized using the software AtomEye [31]. Figure 2a shows the dislocation structure for bulk Cu at a penetration depth *h*_s_ = 15 Å, while Figure 2b–d shows the dislocation structure for Cu at the same penetration depth with a constant porosity equal to 15% and *D*_p_ = 2, 4, and 8 nm, respectively. Figure 2a shows the different stacking faults (atoms in blue) comprised between a leading partial (LP) and trailing partial (LT) dislocations (atoms in dark red). The images reveal that, as *D*_p_ is increased, the dislocation structure resembles more and more the one of bulk Cu (compare Figure 2a,d).

This can be easily understood if one considers that, for the same porosity degree, simulation boxes become more distorted with respect to the initial (bulk) structure as *D*_p_ is decreased. In other words, each pore reduces the coordination number of the atoms located at the surface of the pore, thus reducing the ability to nucleate or propagate a dislocation in its neighborhood. If one calculates which situation has a larger area affected by the reduction in the coordination number, one obtains that, for a single pore, this will be proportional to (*D*_p_/2)^2^. In addition, for a fixed porosity equal to 15%, with *D*_p_ = 2 nm, one needs to draw 6776 pores; for *D*_p_ = 4 nm, 847 pores; and for *D*_p_ = 8 nm, 108 pores. As a consequence, the areas affected by the presence of pores will be 85,150 nm^2^, 42,175 nm^2^, and 21,715 nm^2^ for *D*_p_ = 2, 4, and 8 nm, respectively. Hence, the case with *D*_p_ = 2 nm is the most distorted one (*i.e.*, the one having a higher area affected by the reduction in the coordination number) and, therefore, the one that differs more significantly from the bulk behavior.

Visualization of the dislocation structures also sheds light on understanding why *E*_r_ is reduced with the decrease of *D*_p_. Comparing Figure 2b–d reveals that the pore density (number of pores per unit of volume) increases for a lower *D*_p_, although the overall porosity is constant in the three cases. Thus, for a certain penetration depth, the indentation zone that will encompass more pores will be the one in the simulation box with a smaller pore size, *i.e.*, *D*_p_ = 2 nm.

**Figure 3:**
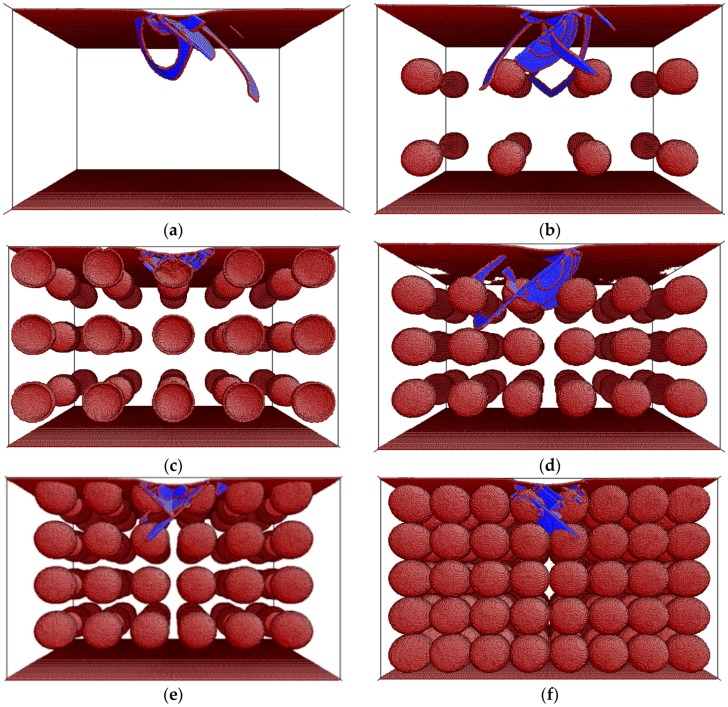
Snapshots showing the dislocation structure at a constant penetration depth (*h*_s_ = 15 Å) of spherical MD indentation simulations on (001) Cu single crystals for: (**a**) bulk; (**b**) *p* = 5%; (**c**) *p* = 10%; (**d**) *p* = 15%; (**e**) *p* = 20%; (**f**) *p* = 45%. The pore size in panels (**b**–**f**) is fixed to *D*_p_ = 8 nm. Dark red color designates atoms with a reduced coordination number.

As aforementioned, the coordination number at the pore edges is reduced with respect to the bulk, and this provides further atomic mobility and lower elastic modulus. Thus, in the simulation box with bigger pores, the probability of having an indentation zone free from porosity and with an *E*_r_ value closer to the bulk one is higher. A similar reasoning explains the reduction of *E*_r_ for the case of constant pore diameter (*D*_p_ = 8 nm) and increase of the porosity degree (Figure 1b,d). This result is shown in Figure 3. Namely, as the porosity increases, the density of pores also increases. Consequently, the indentation zone, for a given penetration depth, is more likely to encompass more atoms with a reduced coordination number, resulting in an overall reduction of *E*_r_.

## 4. Conclusions

In summary, we have shown that the classical expression from Gibson and Ashby [9] can be modified to include the pore diameter and better account for the nanomechanical behavior of nanofoams under nanoindentation. It has also been shown that, for a given porosity level, nanoporous foams with bigger pores behave more similarly to the bulk counterpart than do foams with smaller pores. This effect has been explained bearing in mind the distortion of the atomic structure as a function of pore size, for a given porosity degree. Similarly, for a given pore diameter, increasing porosity level is equivalent to increasing the density of pores per unit volume. Hence, the region of material affected by the indentation zone will encompass more atoms with a reduced coordination number, resulting in a reduction of the sensed elastic modulus. Finally, it should be noted that the results presented here correspond to nanoporous fcc single crystals. Extrapolation to other crystalline structures should be done with caution, since deformation mechanisms acting in each case could differ, to some extent, from those in fcc crystals.
